# Mapping Heat-Related Risks in Northern Jiangxi Province of China Based on Two Spatial Assessment Frameworks Approaches

**DOI:** 10.3390/ijerph17186584

**Published:** 2020-09-10

**Authors:** Minxuan Zheng, Jiahua Zhang, Lamei Shi, Da Zhang, Til Prasad Pangali Sharma, Foyez Ahmed Prodhan

**Affiliations:** 1 Key Laboratory of Digital Earth Sciences, Aerospace Information Research Institute (AIR), Chinese Academy of Sciences (CAS), Beijing 100094, China; zhengminxuan19@mails.ucas.ac.cn (M.Z.); lameis@126.com (L.S.); einsteinzhang@foxmail.com (D.Z.); tilsharma@radi.ac.cn (T.P.P.S.); foyez@bsmrau.edu.bd (F.A.P.); 2 University of Chinese Academy of Sciences, Beijing 100049, China; 3 Department of Agricultural Extension and Rural Development, Bangabandhu Sheikh Mujibur Rahman Agricultural University, Gazipur-1706, Bangladesh

**Keywords:** heat-health risk, spatial risk assessment, heat vulnerability index (HVI), Crichton’s risk triangle, developing countries

## Abstract

Heat-health risk is a growing concern in many regions of China due to the more frequent occurrence of extremely hot weather. Spatial indexes based on various heat assessment frameworks can be used for the assessment of heat risks. In this study, we adopted two approaches—Crichton’s risk triangle and heat vulnerability index (HVI) to identify heat-health risks in the Northern Jiangxi Province of China, by using remote sensing and socio-economic data. The Geographical Information System (GIS) overlay and principal component analysis (PCA) were separately used in two frameworks to integrate parameters. The results show that the most densely populated community in the suburbs, instead of city centers, are exposed to the highest heat risk. A comparison of two heat assessment mapping indicates that the distribution of HVI highlights the vulnerability differences between census tracts. In contrast, the heat risk index of Crichton’s risk triangle has a prominent representation for regions with high risks. The stepwise multiple linear regression zero-order correlation coefficient between HVI and outdoor workers is 0.715, highlighting the vulnerability of this particular group. Spearman’s rho nonparametric correlation and the mean test reveals that heat risk index is strongly correlated with HVI in most of the main urban regions in the study area, with a significantly lower value than the latter. The analysis of variance shows that the distribution of HVI exhibits greater variety across urban regions than that of heat risk index. Our research provides new insight into heat risk assessment for further study of heat health risk in developing countries.

## 1. Introduction

The heatwave is one of the main factors for weather-related illness and death across the world. The frequency, intensity, and duration of heatwaves have increased significantly due to climate change in recent years [1,2]. The elevated temperature ends with immense live loss, e.g., the Chicago heatwave in 1995 [3], the European heatwave in 2003 [4], and the Moscow heatwave in 2010 [5] have led to more than 740, 70,000, and 10,000 deaths, respectively. Thirty percent of the global population is currently exposed to fatal climate conditions for at least twenty days a year. In addition, the threat to human life from extreme heat will increase if global warming continues [6]. Extreme heat events are likely to cause severe human suffering and economic loss, therefore, increasing society’s resilience to these incidents is a critical challenge for government authorities and researchers.

Constructing spatial heat risk indicators is an effective method for quantitative assessment. Several frameworks have been developed to create heat risk indicators, while the frequently used methods are multiplication-based Crichton’s risk triangle framework [7,8,9,10,11,12] and summatory-based heat vulnerability index (HVI) framework [13,14,15,16,17,18,19,20,21,22,23]. Crichton’s risk assessment method has been used to assess flood hazards in the UK [24] and climate-related heat risks [25]. In addition, this method has been frequently applied to heat risk assessment in developing countries in recent years [12,22,26]. The Crichton’s risk triangle framework states that heat risk is a function of hazard, exposure and vulnerability [27,28]: (1) heat hazard describes things that may cause a risk, which can be derived from the historical increasing trend of temperature, the current measured value or the predicted future value; (2) heat exposure commonly refers to the population exposed to heat environment, where the accurate population distribution is needed to calculate exposure to heat [29]; (3) heat vulnerability denotes the vulnerable aspects of the exposed items to a given hazard [28]. The heat risk index is derived from the multiplication of heat hazard, heat exposure and heat vulnerability [9].

The HVI describes the structure of the physical environment and socio-economic conditions and how it responds to risks [30,31]. The approach has been adopted by the IPCC and applied widely, mainly in the country scale and cities with detailed census units and abundant statistical data. The HVI model also includes three aspects; exposure, sensitivity and adaptability. The content described by exposure is similar to the heat hazard index in the risk triangle framework, that is, the extent to which human is threatened by climate change [30]. Sensitivity is also similar to high-temperature exposure; the extent to which people, natural assets or infrastructure respond to climate change and its effects [30]. Adaptivity refers to the ability to reduce loss in the face of extreme temperature events, which is opposite to the heat vulnerability index [30].

In recent years, the methods of heat assessment have been developed based on HVI and Crichton’s risk triangle framework. The common procedure is to select spatial variables that provide some indication of areas at higher or lower risk, followed by integrating these variables through either the unweighted or weighted approaches. The unweighted methods mostly refer to spatial overlay through the Geographical Information System (GIS) technique, while weighted approaches have been introduced in various studies. For example, Zhu [22] used the analytic hierarchy process (AHP) and principal component analysis (PCA) to determine the weight of indicators for HVI. Rinner [21] used the ordered weighted averaging (OWA) multi-criteria analysis to compose indicators for the heat vulnerability assessment of Toronto. In addition, Ho [32] used multi-criteria analysis (MCA) to assign a weight to data layers when constructing heat vulnerability index for Canada. However, the majority of the heat assessment mapping studies utilized PCA or GIS analysis techniques to integrate parameters [16], and few studies have compared the heat assessment mapping results of the GIS overlay and PCA method. Faisal [33] assessed the outcome of urban environmental quality derived from GIS overlay and PCA, and pointed out the difference. According to their research, GIS overlay does not consider the correlation between variables, and the minimum number of components derived in PCA is indeterminable. These differences could cause variation in heat assessment mapping. Given that the recent heat risk assessment studies based on Crichton’s risk triangle framework used spatial overlay method [8,12] while the studies based on HVI framework frequently used PCA methods [26,34], and very few studies have compared the mapping difference between these two patterns; this study was designed to compare the spatial distribution of vulnerability from PCA method over the HVI framework with mapping results of heat risk from GIS overlay method over Crichton’s risk triangle framework.

The senior citizens are recognized to be more sensitive to heat than other populations [31,35,36,37,38,39,40,41], while children [40,42,43,44], the disabled [26,45,46], and groups with low educational levels [31,45,47,48] are considered as the vulnerable groups in heat risk assessment. However, relatively less attention has been paid to the outdoor workers, who are constantly exposed to extreme heat environment and easily obtain work-related injuries [49]. Impaired workers’ health can lead to a decline in human productivity and economic loss [50]; adaptation and preventive measures are needed for these working groups. The literature has shown that apparent temperatures are reliable for assessing the working environment [51], but most heat assessment research studies only consider air temperature and land surface temperature [12,17,52]. In this situation, this study introduced wet bulb globe temperature (WBGT) as an indicator in heat risk assessment. It is a well-established heat index widely used in the assessment of heat stress where high WBGT has been proven to increase the risks of work injuries [53]. WBGT is a combination of the natural wet bulb temperature, the black globe temperature and the air temperature; most formula calculating WBGT include meteorological variables related to temperature, humidity, wind speed and solar radiation. In this study, we calculated WBGT using maximum air temperature and relative humidity. Together with the number of hot days, land surface temperature (LST), and the air quality index (AQI) to provide a comprehensive description of the thermal environment that exposes heat stress on vulnerable population, including outdoor workers. In particular, the data were combined to depict a temporarily extreme heat environment that involves no temporal changes.

Previous studies have focused on the urban settings, while heat assessments in rural area are sparse and inconsistent [54]. Sheridan [53] and Wu [54] have observed greater mortality and vulnerability in response to oppressive heat, in a rural rather than an urban location. At the same time, other studies indicate that urban residents are more vulnerable during heatwave [55,56]. These results suggest that the vulnerability to heat in the rural area is a multifaceted problem that involves factors such as public health infrastructure, heat risk awareness and sociodemographic characteristics [57,58]. However, the understanding of heat health of the rural population in different regions is still in an early stage, especially for developing countries that still have a large proportion of people residing in rural areas and are lack of a systematic geographical and census statistics. Therefore, this study using the northern area of Jiangxi Province in the developing country China as the study area to perform a regional scale research, exploring the high-temperature risk environment of both the urban and rural areas. Considering the data integrity and validity, the year 2015 was used as a bench mark for data collecting.

To summarize, the purposes of this study are: (1) to map heat risk environment using two methodologies developed over HVI and Crichton’s risk triangle frameworks; and (2) to compare the mapping results of two methodologies at the urban and rural areas. Section 2 presents the material and methods; in which we give reasons why each variable was selected as heat risk environment indicator. Section 3 presents the mapping results, as well as explicit explanation, followed by discussion in Section 4, and a conclusion in Section 5.

## 2. Materials and Methods

### 2.1. Study Area

**Figure 1:**
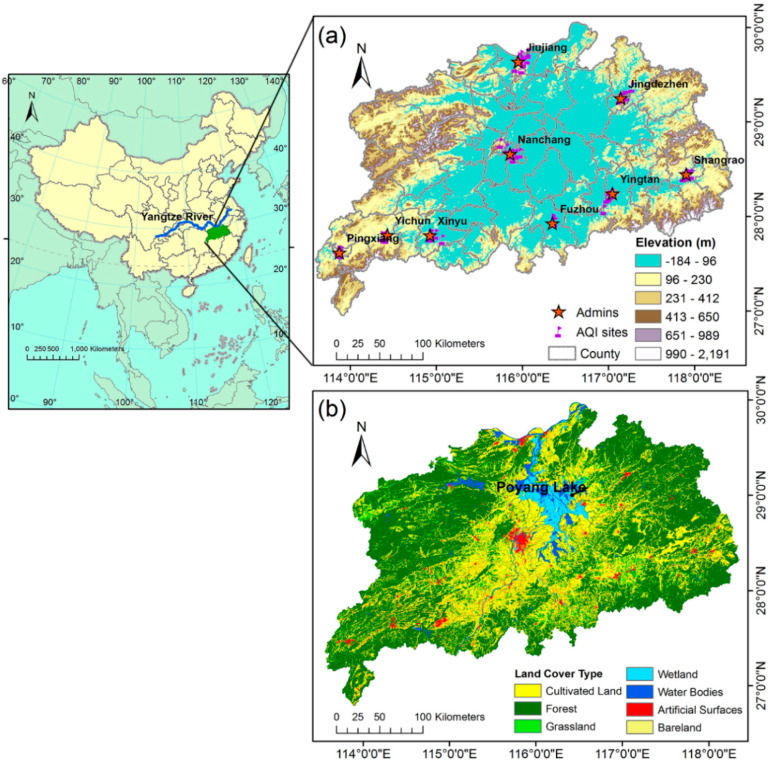
(**a**) elevation and (**b**) land cover type of the study area. Elevation data source: ASTER GDEM (2009), ERSDAC.

**Figure 2:**
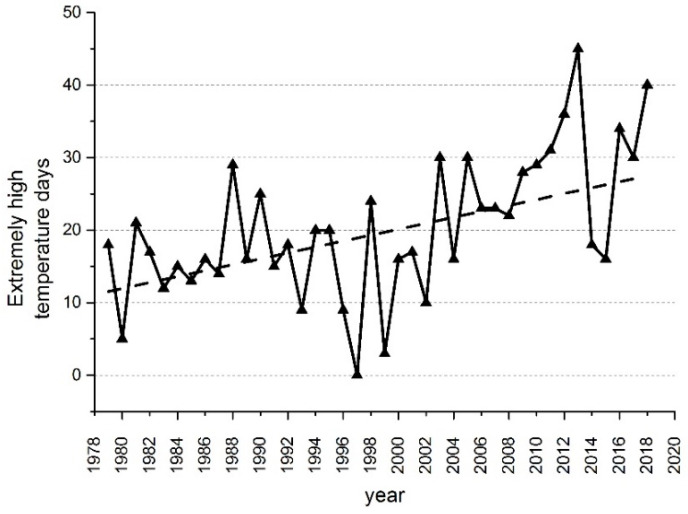
The number of days with temperature above 35 °C in Nanchang from 1979 to 2018. Air temperature data source: National meteorological information center.

Jiangxi Province is located in the middle and lower reaches of the Yangtze River in southeast China (24°29′~30°04′ N, 113°34′~118°28′ E). The region is bounded to the south, east, and west by mountains, with a vast plain in the north-central and wide inner hills (Figure 1a). It belongs to the hot and humid subtropical climate, which is easily affected by extremely high temperatures [59,60,61]. The urban population of Jiangxi Province accounted for 51.62% in 2015. We selected Northern Jiangxi as the study area for two main reasons. First, the Northern Jiangxi Province is classified as an ecological city group in recent years and expected to have rapid development of economy and high population growth. Specifically, the capital city of Jiangxi Province Nanchang with a big increasing trend of high-temperature (>35 °C) days (Figure 2) is located in this area. The exceptionally frequent high-temperature days in Nanchang in 2013 were possibly caused by the great range and strong intensity of west Pacific subtropical high, as well as the large negative precipitation anomalies, which also led to extremely high temperatures in other areas of eastern China in 2013. Nanchang has also been observed to have the fastest increasing trend of extreme heat exposure frequency within the 32 major cities in China [62]. Second, the rising frequency of heatwave in the study area in recent years indicated that it is susceptible to various heat-related health impacts [63,64]. It comprises a total of 64 counties and 10 districts and covers an area of 92,300 square kilometers with 59% cultivated land (Figure 1b), which means that many outdoor workers in this area may be threatened by heat stress.

### 2.2. Data Collection and Preprocessing

#### 2.2.1. Land Surface Temperature

Moderate Resolution Imaging Spectroradiometer (MODIS) land surface temperature (LST) products with a spatial resolution of 1 km were used in this study. Two scenes were selected, one was the MOD11A2 daytime LST (terra satellite with a 10:30 a.m. equator crossing time) from 28 July 2015–5 August 2015; another was the MYD11A2 nighttime LST (aqua satellite with a 1:30 p.m. equator crossing time), from 28 July 2015–5 August 2015. The highest temperature in Nanchang reached 35 °C for those eight consecutive days. Pixels with an accuracy below 2 K were removed, and the data in the study area have no missing values.

#### 2.2.2. Counties’ Air Temperature

Daily maximum temperature of each county was derived from the weather website (https://lishi.tianqi.com/) [65]. The historical weather data provided by this website come from the China Meteorological Administration, and are compiled and released by the website administrator. The daily maximum temperatures of each county in summer during 2015–2018 were obtained through the web crawler, and the annually average number of maximum (>35 °C) daily temperature days were calculated. It can represent the heat environment of the study area in recent years.

#### 2.2.3. Wet Bulb Globe Temperature

The wet bulb globe temperature (WBGT) was calculated based on daily maximum temperature and daily average relative humidity using BioKlima [66]. BioKlima is an universal tool for bioclimatic studies created by Krzysztof Błażejczyk, it could be easily accessed on the website indicated by the reference [66]. A total of 242 sites in the study area was selected using The China Meteorological Assimilation Driving Datasets for the SWAT model (CMADS) V1.1, with a spatial resolution of 0.25° [67]. The final spatial WBGT distribution for the continuous surface was acquired by interpolating eight consecutive WBGT days (corresponding to the MODIS LST time period), using Kriging method and taking their average values in ArcGIS environment.

#### 2.2.4. Air Quality Index Data

Air pollutants increase as temperatures rise [68]; during prolonged heatwaves, the incidence of high concentration of pollutants may reinforce adverse effects on human health [69,70]. Air quality index (AQI) was obtained from the China National Environmental Monitoring Centre for the study region, and the average AQI in each county was calculated. The continuous surface was obtained by the inverse distance weighting (IDW) interpolating method, and the zonal statistics in ArcGIS10.2 (a geographical information system released by the Environmental Systems Research Institute) [71] were used to calculate the mean AQI of each county. The annual average number of days with the highest AQI level in summer from 2015 to 2018 was finally acquired. The calculation methods of AQI were presented in Appendix B.

#### 2.2.5. Socio-Economical and Statistical Data

LandScan global population density spatial distribution product for year 2015 was used to create a population density parameter. The data was derived from global population data released by the US government’s LandScan program (http://web.ornl.gov/sci/landscan/) [72], with a spatial resolution of 1 km. The LandScan product has been developing and continuously updating by the Oak Ridge National Laboratory. Demographic data were derived from the 2010 national census and the 2016 statistical yearbook of Jiangxi Province. Five vulnerable groups were categorized as seniors (≥65 years), young children (≤5 years), disabled people over 16, illiterate people over 15, and people engaged in the nature-based economy. The per capita income of urban and rural residents in each county in 2015 was used to measure the economic situation. The proportion of households without a bathroom facility in a county was used to measure the ability of particular county to cope with extreme heat events.

#### 2.2.6. Proximity to Vegetation

As increased vegetation is associated with decreased extreme heat risk [73], we collected the 16-day enhanced vegetation index (EVI) data (MOD13Q1) at a 250 m resolution for the year 2015 from the Level-1 and Atmosphere Archive & Distribution System Distributed Active Archive Center (https://ladsweb.modaps.eosdis.nasa.gov/search/), and obtained new EVI data through the maximum synthesis method. We finally calculated the statistical mean value of each grid on a 3 × 3 sliding window. The larger value indicates higher vegetation cover in the pixel.

#### 2.2.7. Terrain Data

Inconvenience caused by terrain and transportation may cause difficulties in seeking medical treatment during extreme heat events. We adopted 30 m ASTER GDEM V002 digital elevation model data released by NASA and Japan’s Ministry of Economy, Trade, and Industry (METI) to represent terrain. The slope was calculated using the Slope tool in ArcGIS 10.2, and the relief degree of the land surface (RDLS) was calculated using the focal statistics and raster calculator tool by the following formula [74]:

R = H_max_ − H_min_ (1)

where R refers to the RDLS, H_max_ and H_min_ are the maximum and minimum elevation in the surrounding 7 × 7 pixels respectively. In addition to that, the cubic resampling method was used to resample the above data to 1 km. Highway density was calculated from geographic data, which is also converted into 1 km raster data in ArcGIS10.2.

#### 2.2.8. Proximity to Water

To describe the proximity to water bodies, we used the 2010 Globalland30 land cover classification data provided by the national catalogue service for geographic information. According to the description of landcover class, wetland refers to lands cover with wetland plants and water bodies, and water bodies refer to the water bodies in the land area. We selected both of these landcover types because of their evaporative power to mitigate undesirable human thermal comfort, this could differ them from other landcover areas. The wetland and water bodies were defined as 1 and the other landcover types were 0. The pixels with a value of 1 were averaged in the circular search area of 3 × 3 pixels, and then we resampled the resulting image to 1 km.

### 2.3. Methods

**Figure 3:**
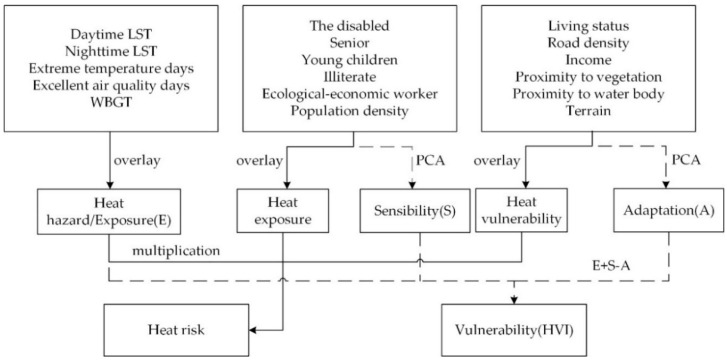
A detailed flowchart of spatial risk assessment methodology.

The method developed over Crichton’s risk triangle is based on the work of Zhang [8], and that developed over HVI followed the common rules introduced by Reid [31]. Open accessed remote sensing and social-economic data were used to derive parameters. Since there is a similarity between the two frameworks, we apply the same variables to the three aspects of each framework. The process is illustrated in Figure 3.

#### 2.3.1. Heat Hazard and Exposure

**Figure A1:**
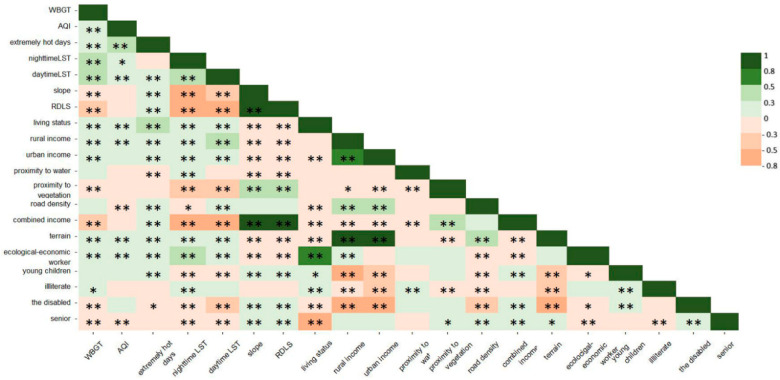
Spearman’s rho nonparametric correlations and significances among the selected variables used in spatial analysis (significance levels: **. Correlation is significant at the 0.01 level (2-tailed). *. Correlation is significant at the 0.05 level (2-tailed).).

Five spatial variables were chosen to calculate heat hazard and exposure index: daytime and nighttime LST, WBGT, the number of extremely hot days, and the number of excellent air quality days. Spearman’s rho non-parametric correlation analysis between each variable was carried out in SPSS software (results shown in Figure A1). The five variables have weak correlation between each other. The Kaiser–Meyer–Olkin (KMO) test coefficient of PCA is less than 0.4, indicating that those variables are not suitable for principal component analysis. Therefore, they were standardized using z-score transformation, such that each variable had a mean of 0 and a standard deviation of 1. The variable indicating excellent air quality days were modified to take the inverse to be summed up with the other four variables with equal weights. The range of the result data was set to 0–100, and then was applied to both frameworks.

#### 2.3.2. Heat Exposure and Sensibility

For the Crichton’s risk triangle, we rasterized the proportion of five vulnerable population data (seniors, young children, disabled people over 16, illiterate, and people engaged in nature-based economy), summed them up, and multiplied the result raster with Landscan data. The heat exposure was obtained by stretching the multiplication result to a range of 0–100.

Five categories of the population, as well as Landscan2015, were standardized using z-score to calculate HVI. They were used as input in the principal component analysis conducted in SPSS 22. After the principal components were obtained, they were rotated to maximize the variance. Retained principal components were weighted by their explained variance respectively, and were finally aggregated to produce the sensitivity index.

#### 2.3.3. Heat Vulnerability and Adaptability

We used four socio-economic variables and four environmental indicators to characterize heat vulnerability index and adaptability: (1) vegetation and water bodies. Vegetation and water bodies have a cooling function that have been reported in many studies [73,75], they are conducive to reducing heat stress and improving the ability to cope with heat risks; (2) transportation accessibility. Mountainous terrain and inconvenient transportation will increase the difficulty of getting access to medical treatment; thus slope, RDLS, and road density were selected as the features; (3) living conditions. Since cooling equipment is related to the vulnerability to the extreme heat events [76], we used the proportion of households without bath facilities in each county as an indicator and (4) per capita disposable income. With a higher income, people have stronger ability to deal with risk, while those who receive low income may experience higher mortality during hot days [77].

For Crichton’s risk triangle, as the correlation between per capita disposable income of urban and rural area as well as the correlation between RDLS and slope were strong (close to 0.8, shown in Figure A1), the mean values of these variables were calculated to obtain two indexes of income and topography. Finally, six variables were added together with equal weights after z-score transformation and were converted to 0–100.

Similarly, the RDLS and slope as a proxy for terrain, and per capita disposable income of urban and rural areas as a proxy for income were integrated into the HVI framework. After z-score standardization, terrain and living condition variables were modified by taking the inverse so that smaller values corresponded to higher adaptabilities [78]. PCA was performed on six variables. After weighting and superimposing, we then stretch the range of the final raster to 0–100 to obtain the adaptability index.

#### 2.3.4. Heat Risk and Vulnerability

After Zhang [8] and Hulley [17], the heat risk index was constructed by the multiplication of heat hazard, heat exposure, and heat vulnerability, and the HVI index was obtained according to the formula below.

HVI = Exposure index (E) + Sensibility index (S) − Adaptability index (A)(2)

We created a stepwise multiple linear regression (SMLR) model, using the zero-order correlation coefficients and partial correlation coefficients to explain HVI with the introduced social and environmental indicators. The variance inflation factor was threshed by 10 to avoid multicollinearity [79], and we chose the model with the highest determination coefficient. Furthermore, to present a better understanding of the mapping results of the two methodologies, we selected the nine main urban regions labeled in Figure 1a to perform further statistical analysis. The extent of the urban region was defined by the 2015 geographical residential area data provided by the national catalogue service for geographic information. After correlating the heat risk index and HVI values using Spearman’s rho correlation, we compared their mean value using Wilcoxon signed rank test, paired sample T-test or Mann–Whitney U test based on the data. We then compared the distribution of heat risk index and HVI value across the nine urban regions using the Kruskal–Wallis test. The post hoc test was also conducted using the Tukey honestly significant difference (HSD) method to identify the urban factors that substantially influence the variance. Statistical analysis was conducted in SPSS 22.

## 3. Results

### 3.1. Heat Hazard and Exposure

**Figure 4:**
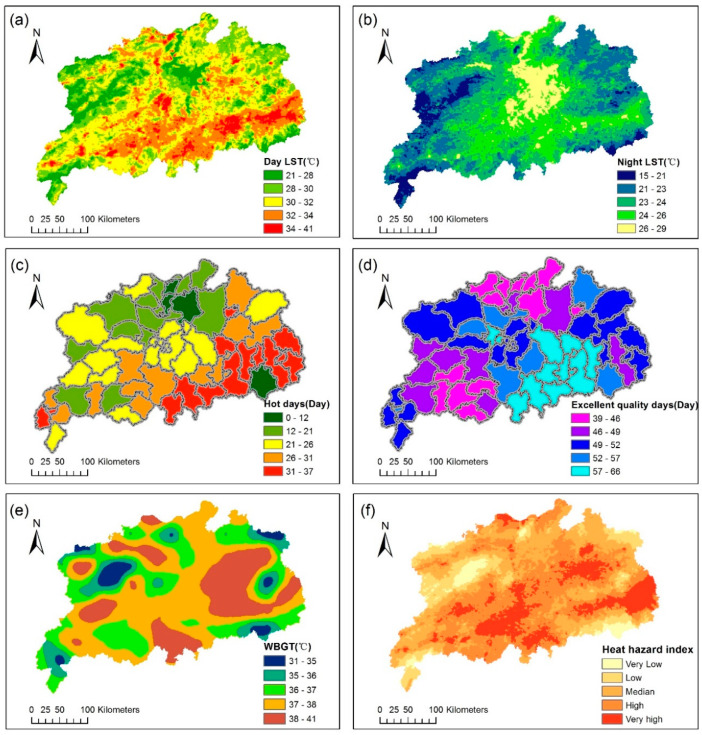
(**a**) daytime land surface temperature (LST); (**b**) nighttime LST; (**c**) the annually average number of days with the highest temperature >35 °C in each county from 2015 to 2018; (**d**) the annually average number of days with the highest air quality index (AQI) level in each county in the summer from 2015 to 2018; (**e**) spatial distribution of 8-day average wet bulb globe temperature (WBGT); (**f**) spatial distribution of heat hazard/exposure index.

As is shown in Figure 4a,b, the spatial patterns of daytime and nighttime temperature exhibit a big difference in the study area. The temperature near Poyang lake appears relatively lower daytime temperature and higher nighttime temperature. The central plain with a large amount of farmland maintains relatively high LST during the day- and nighttime. The spatial distribution of the number of extremely hot days and excellent air quality days are shown in Figure 4c,d. It indicates that not only Nanchang but also many other counties experienced extremely high temperatures. High rainfall occurred during summer in the study area because the monsoon climate [80] (which implies that precipitation amount as well as the occurrence of heavy rainfall events peaks in summer) reduced the air pollutants significantly and led to excellent air quality. This agrees with a national scale research study that reveals that the air quality is good in summer [81], and another research study that suggested that the precipitation is negatively correlated with air pollution [82]. The WBGT was found to be relatively low in some big cities (i.e., provincial capital city Nanchang, Jiujiang city in the north and Pingxiang city in the west), possibly because of the urban dry island effect [83]. The large impermeable surface of cities and the reduction in natural vegetation led to reduced atmospheric humidity and increased vapor pressure deficit, contributing to low relative humidity in cities. In contrast, the extensive rural area at the northeastern part exhibits relatively high WBGT values resulting from high maximum temperature and relative humidity, suggesting that people living or working outside the city in this study area are more likely to be subjected to heat stress.

The spatial distribution of heat risk/exposure was shown in Figure 4f; it is similar to the spatial distribution of daytime LST. In addition, we noticed a high heat risk/exposure index in the northeast part of the study area, possibly affected by WBGT. Through the spatial overlay, the information of the five variables were equally integrated; thereby, the spatial distribution of heat hazard index/exposure depicted study area under extreme temperature environment.

### 3.2. Heat Exposure and Sensitivity

**Figure 5:**
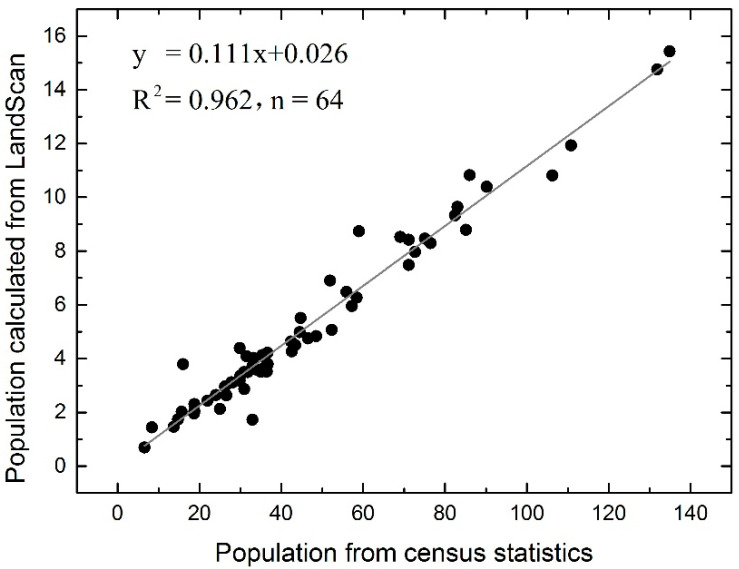
Comparison of total population of each county in Landscan and statistical yearbook. The horizontal axis is the total population at the end of 2015 (ten thousand people), and the vertical axis is the zonal statistics from Landscan after normalization to 0–1.

The correlation analysis between the total population of each county calculated from Landscan and census data were utilized to verify the accuracy of Landscan. Figure 5 shows that the population of each county derived from Landscan is highly consistent with the census statistics, suggesting that Landscan can accurately reflect the spatial distribution of population density.

**Figure 6:**
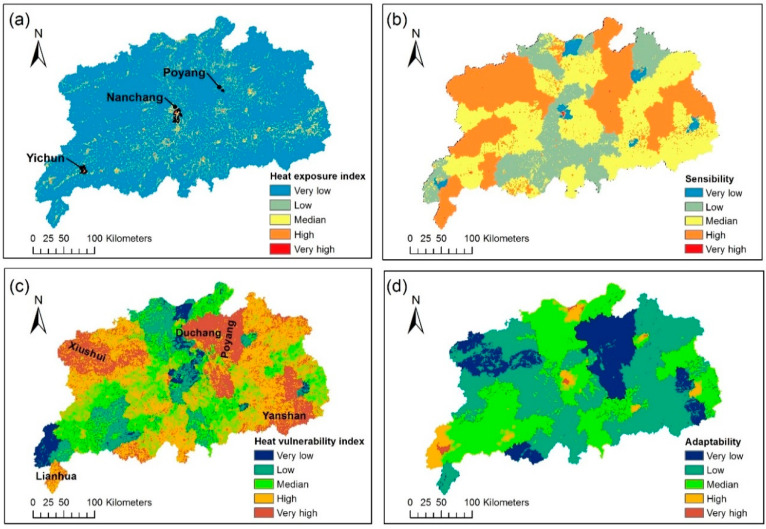
(**a**,**c**): Heat exposure index and Heat vulnerability index of Crichton’s risk triangle; (**b**,**d**): Sensitivity and Adaptability of heat vulnerability index (HVI).

Figure 6a shows the heat exposure in the Crichton’s risk triangle. The population in the study area is mainly distributed in the low elevation area, and most areas have similar heat exposure levels. The area of the highest heat exposure level is small and scattered. It is worth noting that the areas with the highest heat exposure value are in the densely populated settlement rather than in the cities. We selected three residential areas provided by the national catalogue service for geographic information that contained the highest level of heat exposure. They are located in Poyang town, capital city Nanchang, and county Yuanzhou. The population densities are 10,719 km^2^, 9850 km^2^ and 4114 km^2^, and the combined proportions of the five categories of vulnerable populations (children, ecological-economic worker, illiterate, the disabled, senior) are 69.1%, 30.0%, and 54.4% respectively. According to the national county statistical yearbook 2016, Poyang town has the largest resident population in Jiangxi Province. A high heat exposure index is spotted in this area in response to the large proportion of vulnerable people. Therefore, the spatial distribution of the heat exposure index is reasonable.

Under the HVI framework, all variables passed the KMO test and the Barlett hypothesis was rejected. The third principal component with an eigenvalue less than 1 is utilized to ensure that the common degree of variables is larger than 50%. Table 1 shows that the proportion of senior citizens (0.89129), and population density (0.89532) are the dominant variables in the second and third principal components, while the other variables form the first principal component. The data used in the HVI framework methodology have been through z-score standardization. Thus, when conducting the PCA in SPSS, population density, as has obvious spatial characteristics of the data in the sampling points, differed greatly from the other variables, thus, in the whole explanatory variable features small, so is retained in the third principal component. The resultant Figure 6b shows that the highly sensitive areas are partly concentrated in the central district of Nanchang, and dispersed in the municipal cities and suburbs. Overall, the sensitivity index can reflect the area of the highest level of heat exposure in Crichton’s risk triangle.

The standardized population density varies from −0.25 to 62.90. Large areas are identified as being at moderate risk by using natural breaks (Jenks) symbology, which highlights areas with high population density and weakens small differences. While the HVI is a summatory model, the population density has only a partial influence on the sensitivity after PCA. Thus, the spatial distribution of the sensitivity can reflect the overall characteristics of each county.

### 3.3. Heat Vulnerablity and Adaptability

Figure 6c shows the areas with high heat vulnerability value, which include Duchang, Poyang, Xiushui, Lianhua, and Yanshan county, which are related to mountainous terrain, low regional per capita disposable income and backward economic development. The per capita disposable income of Duchang county and Yanshan county are among the last ten of the province’s 100 districts and counties, suggesting that the spatial distribution of heat vulnerability was in line with the actual situation to some extent.

In the HVI framework, adaptability is obtained through PCA based on the rule of Kaiser that the eigenvalues are greater than 1 [84], and the first two principal components in total explained 65.9% of the variances. According to Table 2, areas with high per capita disposable income, high road density and relatively good living conditions demonstrated high adaptability. This result is generally contrary to the spatial distribution of heat vulnerability index, indicating that regions with low heat vulnerability index exhibit high adaptability. Under the HVI framework, PCA was carried out for each variable. Only the main information was retained after PCA, which led to there being less detailed information of HVI’s adaptive distribution map compared with the heat vulnerability index.

### 3.4. Heat Risk and Vulnerability

**Figure 7:**
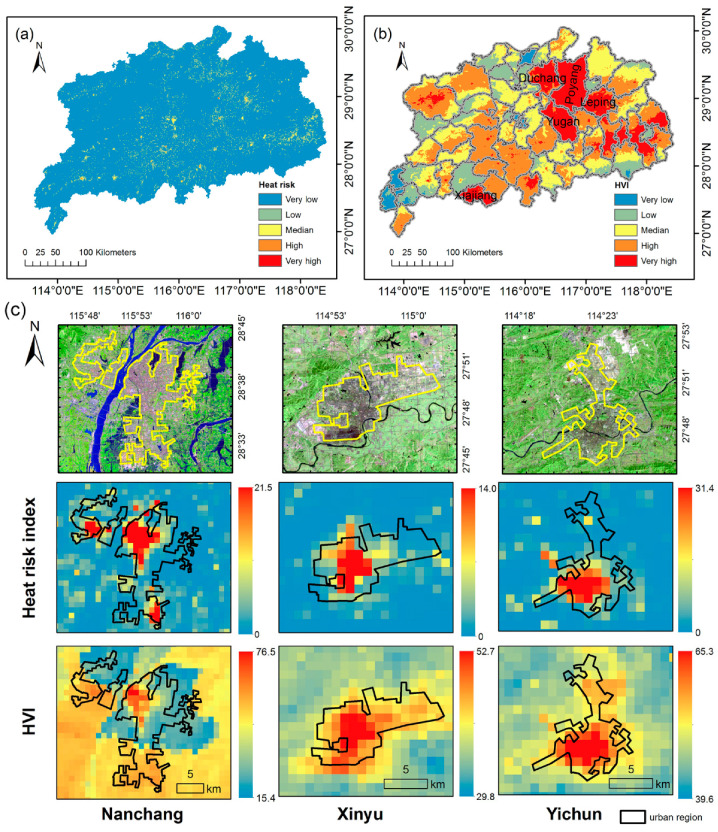
(**a**) spatial distribution of heat risk under Crichton’s risk triangle framework; (**b**) spatial distribution of high-temperature vulnerability index under HVI framework. (**c**) three selected main urban regions (Nanchang, Xinyu and Yichun) and their spatial distributions of heat risk index and HVI.

The spatial distribution of heat risk shows that areas with a medium and high heat risk level are not only in the cities, but also in the suburbs (Figure 7a), e.g., the highest heat risk appeared in Poyang town. The highest value of heat hazard in Poyang town is 67.2, the heat vulnerability is 80.9, and the heat exposure is close to 100, which results in a high heat risk value. Poyang town also has the highest HVI values. As shown in Figure 7b, five counties (Xiajiang, Duchang, Poyang, Yugan, Leping) are classified with an extensive high level of HVI value. In these counties, the sensibility is at a relatively high level and the adaptability is low, thus contributing to the high values of HVI in these counties. In general, the distribution of heat risk is similar to heat exposure, and the spatial distribution of HVI is similar to that of the Adaptability index. Overall, HVI reflects the area with a high or very high level of heat risk in Crichton’s risk triangle, and HVI maintains the differences between counties.

Figure 7c presents an enlarged view of three selected urban regions (Nanchang, Xinyu, Yichun). The urban center shows relatively high heat risk index and HVI value. Influenced by the value of heat exposure, the areas of relatively low population outside the urban area are of low heat risk index. The spatial distribution of HVI tends to have similar values within counties, but the urban center has higher HVI value than that of surroundings in the same county; this is most obvious in Nanchang, which may be caused by the high population density in the urban core area.

**Figure A2:**
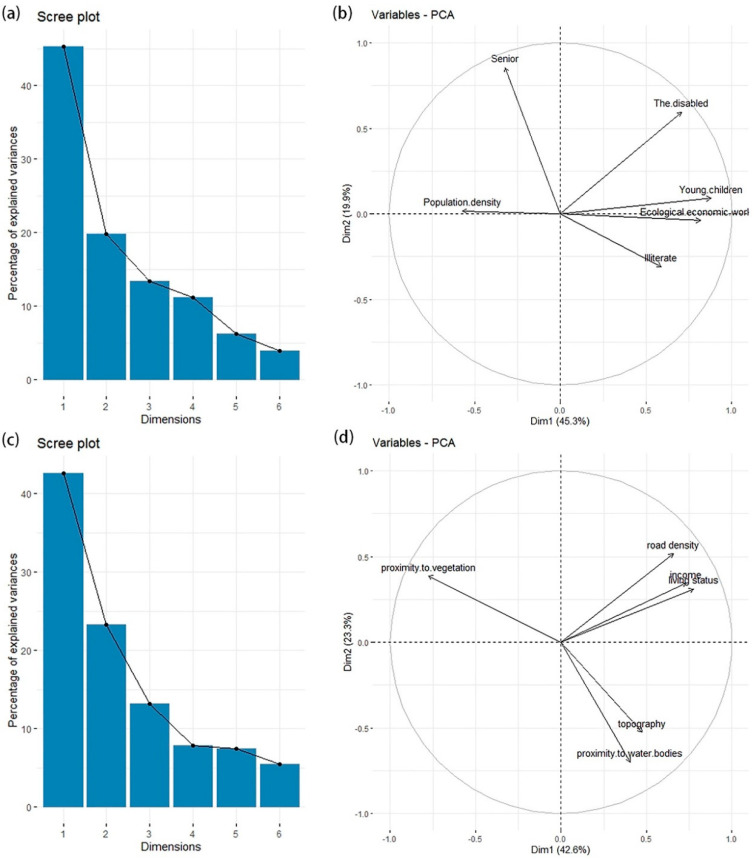
Scree plots and loading factor plots of (**a**) and (**b**) population variables; (**c**) and (**d**) heat vulnerability and adaptation variables.

The SMLR model with the highest determination coefficients included all the introduced variables which showed no multicollinearity. The zero-order correlation coefficients and partial correlation coefficients between HVI and variables are shown in Table 3. Variables with the relatively higher correlation were living condition (−0.782) and the proportion of people engaged in the nature-based economy (0.715), suggesting that the housing conditions and the outdoor workers have the greatest influence on HVI. The disabled, illiterate and young children show similar zero-order correlation coefficient while the seniors show no correlation. However, when the influence of the other variables was in control, the seniors show moderate partial correlation coefficient (0.565) with HVI. This could be partly explained by the difference between the characteristics of population categories displayed in Figure A2. The senior has a dominant loading in the second principal component, while the others are grouped in the first principal component. Besides the senior, the differences between the correlation coefficient and partial correlation coefficient of population density (−0.152 and 0.860), excellent air quality days (0.192 and −0.881), daytime LST (−0.055 and 0.833), nighttime LST (−0.102 and 0.793), and extremely high temperature days (0.238 and 0.862) are quite large, which implies that these variables are greatly influenced by other variables. Some variables, such as the days with the highest air quality level, proximity to water bodies, and topography, have low correlations; while proximity to vegetation is positively correlated with HVI.

The results show that proximity to vegetation is positively correlated with HVI and negatively correlated with the first principal component in Section 3.3; inconsistent with the preliminary hypothesis that the closer the distance to vegetation and water bodies, the lower the vulnerability to extreme heat. An explanation for this situation is that in the study area, the area of cultivated land, forest and grassland occupied 91% of the land cover, the remote area has especially widespread agricultural land and forests. The remote area is related to backward economy and insufficient infrastructure, thus making the proximity to vegetation parameter display consistent relation with HVI and inverse relation with other parameters when calculating principal component.

**Figure 8:**
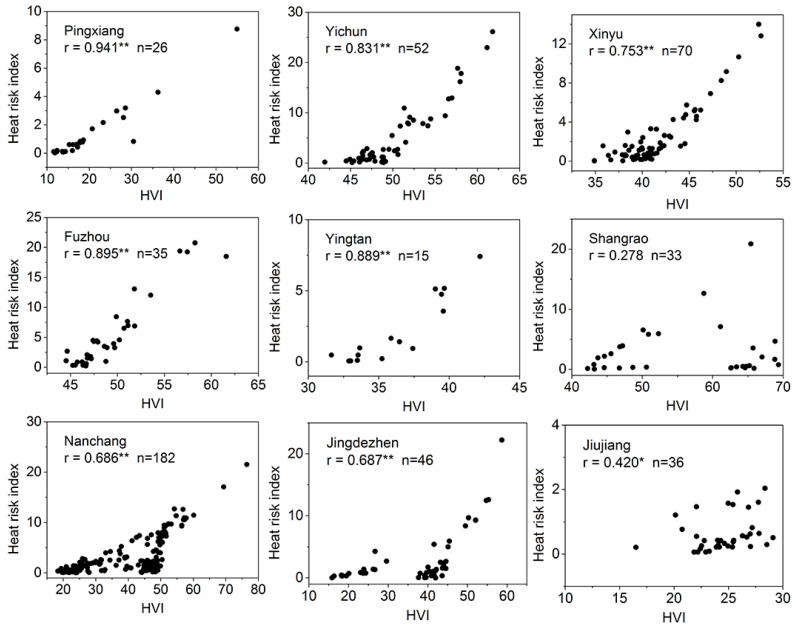
Spearman’s rho correlation coefficients of the nine main urban regions in the study area. ** means correlation is significant at the 0.01 level (2-tailed), * means correlation is significant at the 0.05 level (2-tailed).

In Figure 8, the heat risk index revealed significant correlations with the HVI value in the selected urban regions, except for Shangrao. Some urban regions included a large proportion of low heat risk values that are close to 0. Visually, after dropping them, the data could be fitted by exponential function. The statistical results (Table A3) show that the mean of the heat risk index is significantly lower than HVI value in the nine urban regions.

**Figure 9:**
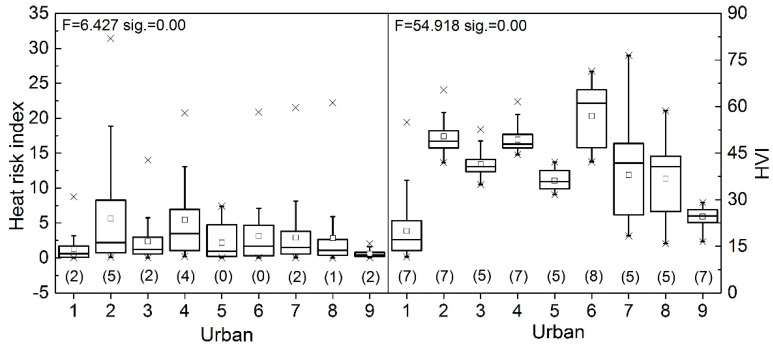
Comparing nine main urban areas (horizontal indexes 1–9 refer to Pingxiang, Yichun, Xinyu, Fuzhou, Yingtan, Shangrao, Nanchang, Jingdezhen and Jiujiang). The boxes represent the 25th to the 75th percentile; the crosses are the maximum and minimum values. The number in bracket is the quantity of urban regions that are significantly different from the urban of the corresponding horizontal index. The significance level is 0.05.

As shown in Figure 9, most of the heat risk values within the nine urban regions are below 30, while the range of HVI value within urban regions is from about 10 to 90. Yichun (Urban 2) has the highest heat risk value among urban regions, possibly because of high heat vulnerability index, while the highest HVI value exhibited in Nanchang (Urban 7) may be due to high exposure value. The Kruskal–Wallis tests (not shown here) reveal that both of the heat risk index and HVI have significantly different distributions across the nine urban regions and the results of Post Hoc test show that the difference of HVI distribution (F = 54.918, *p* < 0.05) are substantially larger than that of heat risk index (F = 6.427, *p* < 0.05). Yichun has the most special heat risk index distribution (significantly different from five urban regions), while Shangrao (Urban 6) has the most special HVI distribution (significantly from eight urban regions).

## 4. Discussion

This study presents a comparison of the heat health risk of the population in northern Jiangxi, China by spatial overlay and PCA methods, based on Crichton’s risk triangle and HVI framework. In Crichton’s risk triangle, as there is no commonly acknowledged standard weight for each parameter, all of them were equally weighted, and the area of highest heat risk was highlighted. In addition, the spatial distribution of heat risk is similar to that of the index with a great variation range, which is consistent with previous studies [8]. PCA used in the HVI framework is more objective and can reduce the impact of population density with a large degree of dispersion. Although both of the methodologies pointed out the same highest risk area and are strongly correlated, heat risk index features relatively lower values than HVI within urban regions and a smaller variety of spatial distribution across urban regions.

The study area reflects the well documented urban heat island (UHI) phenomenon, which results in a conurbation being warmer than the surrounding rural areas [85,86]. As a planned economic development area, northern Jiangxi may continue to expand the city scale. Cities, as the economic and cultural focal point with high population density, are prone to severe environmental impacts, which accordingly means that they are particularly vulnerable to climate change [87]. Both methodologies show the inner-city areas with high population density have high heat risk or HVI value, which is consistent with previous studies in the Yangtze river delta and Chongqing region, as well as in the USA [8,9,31,88]. Moreover, the spatial distribution of HVI shows that the non-central city area has a lower vulnerability; this could be attributed to better infrastructure and a relatively low proportion of the vulnerable population. Similar to other developing countries, there are densely populated areas outside the city which exhibits high heat risk [89]. The highest heat risk and HVI value that occurred were observed in a suburban community in Poyang. Census statistics have shown that the abnormally high population density and relatively low level of economic development lead to this situation, which has been reported in multiple studies that increased heat risks are related to increased population density, both in urban and rural areas [88,90]. Other vulnerable counties were rural, clustered in the northeast of the study area, with living status, vulnerable population and road density as the primary drivers. This requires local residents to raise awareness of heat risk prevention and local government to strengthen the construction of public infrastructure, which is conducive to the prevention of heat hazards.

People engaged in a nature-based economy are closely associated with HVI. Meanwhile, although inaccuracy originates from the calculation of WBGT that wind speed in 2 m and solar radiation were not concerned, making the value of WBGT unable to represent the real outdoor thermal stress, the spatial distribution of the exposure index that is integrated with WBGT shows that the rural area exhibits much higher values. As many economic-economy and outdoor workers are working in rural areas and their intense physical labor, they are more likely to be threatened by severe heat stress. Xiang [91] has found that excessive heat stress could lead to occupational heat-induced illness and an increase in medical costs and work days lost. Moreover, George Maier et al. [18] have used AT as a variable to evaluate the vulnerability of Georgia, United States under heat stress based on the framework of HVI, and found that the death rate of heat stress weather was 13.4% higher than that of non-heat stress weather. Hence, WBGT explored in this research could serve as a good reminder that highlight the heat exposure in rural areas, as well as the heat-health related burden of the outdoor worker.

Land surface temperature or extreme temperature days show weak zero-order correlation with HVI. Actually, most of the fine scale raster-based data have relatively low zero-order correlation with HVI, which might be attributed to the differences in scale and resolution between the socioeconomic and environmental data. Despite this, the partial correlation suggested the strong influence on HVI from hot environment depicted by land surface temperature and extremely hot days. Moreover, as temperature extremes and variability will remain important determinants of health [92,93], spatial distribution of HVI emphasizes the area with high risk and reveals great variation across urban areas, and thus could provide suggestions for heat alerts and developing emergency interventions.

The strong correlation between HVI and heat risk index in most urban regions (8/9) proves that the mapping results of the two methodologies have good consistency in urban regions and can reflect the areas of high heat-health risk. It is predictable that the heat risk index values within urban regions are significantly lower than HVI values, as can be seen in the mapping result of the whole study area. Very few areas reached the high level of risk, and most of the heat risk index values are distributed in lower tail. In contrast, the spatial distribution of HVI could reflect the heterogeneity across counties, making it exhibit a larger variety across urban regions.

There are still some research gaps in this paper; (1) data of preexisting health concerns that denote vulnerability to heat conserved in the Chinese center for disease control and prevention, such as cardiovascular disease or psychiatric disorders, are not currently accessible [26], adding difficulty to the verification of the results. Due to the different availability of data, the time range of variables was inconsistent, and the statistical indicators of each county in the study area were not unified, limiting the construction of indicators [32,94]. Some variables, such as home air conditioning, which is a strong protective factor against extreme heat events, have not been proxies in local statistics and are not considered in this research; (2) a typical period of high temperature is selected in this paper for the assessment of extreme temperature. Some studies have emphasized the spatial-temporal change assessment of heat risk in developing countries—this may be considered in future study—and (3) people’s ability to adapt to extreme heat environment will change, and extreme heat events may increase with global anthropogenic climate change. This involves many socio-economic, individual behavior and environmental change factors and needs further consideration [95].

In the future, we hope to use a wider range of environmental and socio-economic data as well as remote sensing data of more explicit resolution, such as Sentinel or Ecosystem Spaceborne Thermal Radiometer Experiment on Space Station (ECOSTRESS) thermal infrared sensor data to explore areas of high heat risk at local scale and during different time periods. Moreover, with fined-scale population statistics available, risk assessments aimed at particular categories of vulnerable population are in desideratum.

## 5. Conclusions

The spatial heat-risk assessment is still a great concern for researchers and governments. Along with the increasing number and severity of excessive heat weather, the needs for research into risk assessment has been highlighted. Regions located in subtropical climate with increasing warming trends like northern Jiangxi suffer an even higher health risk. This study thus explored the spatial variation of heat-health risk in northern Jiangxi, based on two methodologies. These methodologies are developed over Crichton’s risk triangle framework and HVI framework separately; GIS spatial overlay and PCA method are used to integrated various socio-economic and environmental indicators. After mapping the heat-health risk at regional scale, we further explored the quantitative features of heat risk index and HVI values in the nine main urban regions in the study area. Given that regional health authorities in many countries have developed heat health emergency plans for targeting interventions at high-risk populations during future events, our research using classic methods, easily accessible raster data and statistical information, could provide references for heat assessment model construction and hot weather planning in areas with limited open access data.

## Figures and Tables

**Table 1:** Principal component (PC) coefficients (loadings) for the sensibility variables for the three retained varimax-rotated principal components.

*n* = 620	PC1	PC2	PC3
Children	0.85521	0.00353	−0.2676
Ecological-Economic Worker	0.73492	−0.1261	−0.3392
Illiterate	0.72488	−0.3445	0.2007
The Disabled	0.67725	0.51603	−0.3619
Senior	−0.1625	0.89129	0.18427
Population Density	−0.1765	0.11747	0.89532

**Table 2:** Principal component (PC) coefficients (loadings) for adaptation variables for the two principal components.

*n* = 620	PC1	PC2
Living Status	0.77593	0.31217
Income	0.73143	0.34529
Road Density	0.65592	0.51723
Proximity to Water	0.40653	−0.6998
Topography	0.47436	−0.5256
Proximity to Vegetation	−0.7752	0.38734

**Table 3:** Stepwise multiple linear regression correlation coefficient and the partial correlation coefficient between HVI and introduced variables.

*n* = 315	Correlation			Correlation	
	Zero-Order	Partial		Zero-Order	Partial
Living Status	−0.782	−0.843	young children	0.668	0.530
Road Density	−0.669	−0.944	nighttime LST	−0.102	0.793
WBGT	0.452	0.863	proximity to vegetation	0.402	0.699
Income	−0.661	−0.836	the disabled	0.532	0.647
Population Density	−0.152	0.860	illiterate	0.530	0.609
Daytime LST	−0.055	0.833	ecological-economic worker	0.715	0.276
Senior	−0.256	0.565	terrain	−0.033	−0.226
Excellent Air Quality Days	0.192	−0.881	proximity to water body	−0.172	0.187
Extreme Temperature Days	0.238	0.862	-	-	-

## Appendix B

Here, we present the information about the calculation of the air quality index. Source: Ministry of environment protection of China. Air quality index (AQI) is an indicator to measure how polluted the air or the environment in a region. According to the “technical regulation on ambient air quality index” released by ministry of environment protection of China in 2012, the calculation of AQI is dependent on six pollutants, namely, PM_2.5_, PM_10_, CO, SO_2_, NO_2_, O_3_. AQI was calculated from the individual air quality index (IAQI) of each pollutant by the following formula:

(A1) $${\mathrm{AQI}\;=\;\max\{\mathrm{IAQI}}_{1}{,\mathrm{IAQI}}_{2}{,\mathrm{IAQI}}_{3}{,\ldots ,\mathrm{IAQI}}_{n}\}$$

IAQI was calculated according to the concentration of each pollutant and a threshold table (Table A1) by the following formula:

(A2) $${\mathrm{IAQI}}_{P}\;=\;\frac{{\mathrm{IAQI}}_{\mathrm{Hi}}{-\mathrm{IAQI}}_{\mathrm{Lo}}}{{\mathrm{BP}}_{\mathrm{Hi}}{-\mathrm{BP}}_{\mathrm{Lo}}}\left(C_{P}{-\mathrm{BP}}_{\mathrm{Lo}}\right){\;+\;\mathrm{IAQI}}_{\mathrm{Lo}}$$

where IAQI_P_ represents the IAQI of the pollutant P. C_P_ is the concentration of pollutant P, BP_Hi_ and BP_Lo_ are the high and low value of the threshold closest to C_P_. IAQI_Hi_ and IAQI_Lo_ are the individual air quality indices corresponding to the BP_Hi_ and BP_Lo_.

The threshold of Individual Air Quality Index and the concentration of pollutant item and classification threshold for Air Quality Index are listed as Table A1 and Table A2.

**Table A1:** Threshold of individual air quality index and concentration of pollutant item.

IAQI	SO_2_	NO_2_	PM_10_	CO	O_3_	PM_2.5_
	μg/m^3^	μg/m^3^	μg/m^3^	μg/m^3^	μg/m^3^	μg/m^3^
0	0	0	0	0	0	0
50	50	40	50	2	100	35
100	150	80	150	4	160	75
150	475	180	250	14	215	115
200	800	280	350	24	265	150
300	1600	565	420	36	800	250
400	2100	750	500	48	>800	350
500	2620	940	600	60	>800	500

Note: The threshold values of SO_2_, NO_2_, PM_10_, PM_2.5_, and CO are the 24 h average values, while the threshold values of O_3_ have an 8 h average value.

**Table A2:** Classification threshold for air quality index.

AQI	Level	Description
<50	I	Excellent
0	II	Good
50	III	Mild pollution
100	IV	Medium pollution
150	V	Heavy pollution
200	VI	Severe pollution

## Appendix C

**Table A3:** Comparing the mean of heat risk index and HVI values in nine main urban regions (urban 1–9 refer to Pingxiang, Yichun, Xinyu, Fuzhou, Yingtan, Shangrao, Nanchang, Jingdezhen and Jiujiang). Shapiro–Wilk method was used to test the normality; Wilcoxon signed rank test, paired-sample T-test and Mann–Whitney U test were used according to the condition of the samples. The significance value is 0.05.

Urban 1	Test of Normality			Wilcoxon Signed Rank Test	
	statistic	df	sig.	standardized test statistics	sig.
HVI-heat risk index ^1^	0.79	26	0.00	4.46	0.00
Urban 2	Test of normality			Wilcoxon signed rank test	
	statistic	df	sig.	standardized test statistics	sig.
HVI-heat risk index	0.88	52	0.00	6.28	0.00
Urban 3	Test of normality			Wilcoxon signed rank test	
	statistic	df	sig.	standardized test statistics	sig.
HVI-heat risk index	0.96	70	0.01	7.27	0.00
Urban 4	Test of normality			Wilcoxon signed rank test	
	statistic	df	sig.	standardized test statistics	sig.
HVI-heat risk index	0.86	35	0.00	5.16	0.00
Urban 5	Test of normality			Paired sample T-test	
	statistic	df	sig.	t	sig.
HVI-heat risk index	0.98	15	0.94	94.34	0.00
Urban 6	Test of normality			Mann-Whitney U test	
	statistic	df	sig.	standardized test statistics	sig.
HVI	0.88	33	0.00	6.98	0.00
heat risk index	0.70	33	0.00		
Urban 7	Test of normality			Wilcoxon signed rank test	
	statistic	df	sig.	standardized test statistics	sig.
HVI-heat risk index	0.87	182	0.00	11.70	0.00
Urban 8	Test of normality			Wilcoxon signed rank test	
	statistic	df	sig.	standardized test statistics	sig.
HVI-heat risk index	0.78	46	0.00	5.91	0.00
Urban 9	Test of normality			Paired sample T-test	
	statistic	df	sig.	t	sig.
HVI-heat risk index	0.97	36	0.33	6.72	0.00

^1^ HVI-heat risk index: difference between the HVI value and heat risk index value.
